# Acute onset of intracranial subdural hemorrhage five days after spinal anesthesia for knee arthroscopic surgery: a case report

**DOI:** 10.1186/1752-1947-6-75

**Published:** 2012-03-06

**Authors:** Tetsuo Hagino, Satoshi Ochiai, Yoshiyuki Watanabe, Shinya Senga, Masanori Saito, Hirofumi Naganuma, Eiichi Sato, Hirotaka Haro

**Affiliations:** 1 The Sports Medicine and Knee Center, Kofu National Hospital, 11-35 Tenjin-cho, Kofu, Yamanashi 400-8533, Japan; 2 Department of Orthopedic Surgery, Interdisciplinary Graduate School of Medicine and Engineering, University of Yamanashi, 1110 Shimokato, Chuo-city, Yamanashi, 409-3898, Japan; 3 Department of Neurosurgery, Kofu National Hospital, 11-35 Tenjin-cho, Kofu, Yamanashi 400-8533, Japan

## Abstract

**Introduction:**

Spinal anesthesia is a widely used general purpose anesthesia. However, serious complications, such as intracranial subdural hemorrhage, can rarely occur.

**Case presentation:**

We report the case of a 73-year-old Japanese woman who had acute onset of intracranial subdural hemorrhage five days after spinal anesthesia for knee arthroscopic surgery.

**Conclusion:**

This case highlights the need to pay attention to acute intracranial subdural hemorrhage as a complication after spinal anesthesia. If the headache persists even in a supine position or nausea occurs abruptly, computed tomography or magnetic resonance imaging of the brain should be conducted. An intracranial subdural hematoma may have a serious outcome and is an important differential diagnosis for headache after spinal anesthesia.

## Introduction

Spinal (subarachnoid) anesthesia is a widely used general purpose anesthesia. However, serious complications, such as intracranial subdural hemorrhage, can rarely occur [1]. The incidence of intracranial subdural hemorrhage after spinal anesthesia has been reported to be one in 500,000 [2]. In a literature review, 35 cases were identified and most had a chronic course with onset after a postdural puncture headache [1]. We report a case of acute onset of an intracranial subdural hematoma without trauma five days after spinal anesthesia.

## Case presentation

A 73 year-old Japanese woman underwent arthroscopic surgery of the knee for a torn medial meniscus. She had a surgical history of excision of a right frontal meningioma 11 years ago. She was receiving medications for hypertension and hyperlipidemia. She had no history of trauma, headache or coagulation abnormalities.

From around four years before presentation to our center, our patient started to experience right knee pain and was treated at her local hospital with intra-articular injections of hyaluronic acid and physical therapy. She was referred to our center because of deterioration of pain and a catching sensation on the medial side of her right knee joint. Examinations at presentation detected pain from the medial side to the popliteal region of her right knee joint, a restricted range of motion to -10 degree extension and 110 degree flexion and a positive McMurray test. A plain radiograph showed findings of osteoarthritis. Magnetic resonance imaging (MRI) revealed a degenerative tear of the medial meniscus. Arthroscopic surgery was scheduled and our patient was admitted.

### Anesthesia and intraoperative course

Spinal anesthesia was conducted by puncturing the L4-5 space with a 26-gauge spinal needle and injecting 2.3 mL of 0.5% isobaric bupivacaine. The puncture was successful on the first attempt, and the course of anesthesia was without incident. During surgery, arthroscopic examination showed wear of the articular cartilage and a degenerative tear of the medial meniscus; partial excision of the medial meniscus was conducted. Her intraoperative vital signs were normal and the surgery was completed uneventfully. The operation time was 87 minutes. At discharge from the operation room, her sensory block region was below the 10^th ^thoracic vertebra.

### Postoperative course

**Figure 1:**
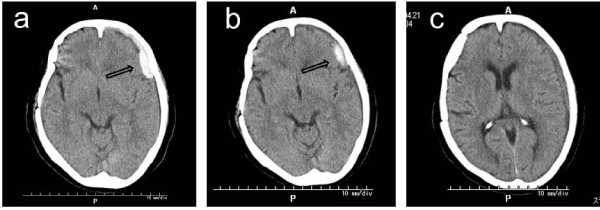
**CT findings**. **(A) **Immediately after onset, a subdural hematoma is observed extending from the left frontal to temporal region (arrow). **(B) **On day 12 after onset, the volume of the hematoma is reduced and density is lowered (arrow). **(C) **One month after onset, further reduction in the volume of the hematoma is observed.

Our patient used a wheelchair one day after the operation and started physiotherapy on her third postoperative day. There were no symptoms of a postdural puncture headache, and the postoperative course was uneventful. On the fifth postoperative day, our patient started to have a headache with vomiting at 6 a.m. while defecating in the toilet. The severe headache persisted even in a supine position, and her blood pressure was 232/103 mmHg. Pentazocine was administered for the headache. Our patient appeared somnolent but showed no definitive paralytic symptoms. At 9 a.m., a computed tomographic (CT) scan was performed, which showed a subdural hematoma from her left frontal to temporal region. The greatest thickness of the hematoma was 1 cm, and a slight midline shift was observed (Figure 1A). MRI performed consecutively did not show an aneurysm, only the subdural hematoma. The size of the hematoma did not change on CT examinations performed six hours and 24 hours later.

Our patient was treated conservatively with blood pressure control, administration of a hemostatic agent and bed rest. Her headache was resolved after seven days of rest. On day 12 after onset, a CT scan showed that the hematoma had reduced in volume and in density (Figure 1B), indicating hematoma absorption. Rehabilitation was restarted from the 13^th ^postoperative day. A CT scan one month later showed further reduction in the volume of the hematoma (Figure 1C). On the 49^th ^postoperative day, our patient had no subjective symptoms and was discharged.

## Discussion

Postdural puncture headache is the major complication observed after spinal anesthesia. The symptom may be aggravated by a change in posture and is accompanied by other symptoms such as nausea, vomiting, and vertigo [3]. The headache is postulated to be caused by tension in the intra- and extracranial nerves and blood vessels associated with cerebrospinal fluid leak, and vasodilation due to the compensatory increase of intracranial pressure [4]. Intracranial subdural hemorrhage is also reported to occur as a result of dilatation and traction of the thin bridging vein in the wall, resulting in rupture [5]. Furthermore, Rocchi *et al. *[6] reported a case of intracranial and intraspinal hemorrhage after spinal anesthesia. They suggested that multiple attempts for spinal anesthesia most likely cause rupture of the spinal vessels, either directly or indirectly by inducing differential pressure changes between the cerebrospinal fluid and intravascular spaces; however, definite mechanisms are not completely understood. In the present case, the headache occurred while our patient was in the toilet on day 5 after surgery. It is possible that low cerebrospinal fluid pressure without headache already existed after anesthesia, and straining during defecation increased the venous pressure, resulting in rupture of the vein and the onset of subdural hemorrhage.

Nakanuno *et al. *[7] studied 69 cases of intracranial subdural hematoma after dural puncture for the purpose of anesthesia, diagnosis or treatment, and reported that the major onset symptom was non-postural headache often associated with neurological symptoms such as consciousness disorder, vomiting, hemiplegia and diplopia. They classified the duration of headache until subdural hematoma into three patterns: a headache that occurred early (within four days) after dural puncture and persisted with subsequent onset of subdural hemorrhage; a headache that occurred early after dural puncture that disappeared or was alleviated transiently but reappeared and was aggravated, followed by onset of subdural hemorrhage; and a headache that did not occur early after dural puncture but appeared later with onset of subdural hemorrhage. In their study, the first pattern was found in 47% (33 cases), the second in 44% (30 cases) and the third pattern in 6% (4 cases); 3% (2 cases) were unknown. Most of the cases had a headache early after the dural puncture. The third pattern, with acute onset and no early stage headache, as was observed in the present case, was rare.

Amorim *et al. *[1] reported two cases of intracranial subdural hematoma after spinal anesthesia and reviewed a total of 35 cases in the literature, including their own cases. The duration from symptom onset to diagnosis ranged from four hours to 29 weeks (mean 22 days). Only four of 35 cases were diagnosed within one day of the appearance of acute symptoms, and onset was on the day of anesthesia in all four cases. In the other 31 cases, the headache preceded the development of intracranial subdural hemorrhage and the diagnosis was established after a prolonged period. Four of the 35 reviewed cases developed neurologic sequelae and four died. None of the reviewed cases showed a similar course to our case: no early symptoms after spinal anesthesia and acute onset after the fifth postoperative day. Therefore, attention has to be given to the possibility that intracranial subdural hematoma may develop acutely without preceding symptoms in the early post-anesthesia stage.

## Conclusion

In patients with a persistent headache following dural puncture, where the headache persists even in a supine position or nausea occurs abruptly, CT or MRI of the brain should be performed. Am intracranial subdural hematoma may have a serious outcome and is an important differential diagnosis for headache after spinal anesthesia.

## Consent

Written informed consent was obtained from the patient for publication of this case report and any accompanying images. A copy of the written consent is available for review by the Editor-in-Chief of this journal.

## Competing interests

The authors declare that they have no competing interests.

## Authors' contributions

TH, SO, YW, SS, MS, HN, ES and HH analyzed and interpreted our patient data regarding the clinical course, surgery and outcome. TH was a major contributor in writing the manuscript. All authors read and approved the final manuscript.
